# Three-dimensional reconstruction system in pulmonary broncho vascular surgery using AI

**DOI:** 10.1016/j.ebiom.2023.104495

**Published:** 2023-02-26

**Authors:** Seifedine Kadry

**Affiliations:** a Artificial Intelligence Research Center (AIRC), Ajman University, Ajman, 346, United Arab Emirates; b Department of Electrical and Computer Engineering, Lebanese American University, Byblos, Lebanon; c Department of Applied Data Science, Noroff University College, Kristiansand 4612, Norway

Modern clinics rely heavily on medical imaging for diagnosis and treatment. Therefore, the reconstruction of medical images is one of the most fundamental components of medical imaging, which aims to acquire high-quality images for clinical use at minimal cost and risk.

Phantoms or imaging phantoms are scientific devices widely used in medical imaging to test and optimize imaging devices without exposing humans to radiation.^1^ A phantom based on human anatomy is an object made of materials resembling a real organism's tissues. Anthropomorphic phantoms can be used for various tasks because they are available and look like real patients. To maximize the use of radiation, surgical planning, and image reconstruction techniques, anthropomorphic phantoms are used instead of imaging multiple patients. Thoracic surgery refers to operations on organs in the chest, including the heart, lungs, and esophagus.^2^, ^3^

Medical imaging can be enhanced with these phantoms that simulate the pulmonary broncho-vascular system. Therefore, it would be possible to diagnose several pathologies earlier and more accurately and perform faster and more precise interventions. In addition, this would reduce invasive surgical procedures and their associated costs.^4^

Deep learning has gained momentum over the past decades due to significant advances in algorithm development and computational power. In 2019, GE Healthcare approved a reconstruction algorithm based on deep learning reconstruction (TrueFidelity, Waukesha, WI). Additionally, vendors develop their algorithms for deep learning reconstruction. For example, the DLR algorithm has been trained using high-dose and low-dose FBP datasets from phantom and patient studies to suppress image noise without compromising image quality. According to a technical white paper,^5^ the DLR algorithm has been trained on phantom and patient datasets to reduce image noise without compromising image quality. As a result, DLR can improve image quality in previously challenging areas, like low-dose imaging and obesity scanning.

Using semi-automated tools (such as Mimics®, OsiriX, and 3D slicer), lung 3D reconstruction models can be simulated, pulmonary segment divisions clarified, and lesion locations determined. Although these systems are promising, their professional skill requirements, manual annotations, and high-time requirements may curb their widespread adoption in clinical practice. Using AI techniques could improve clinical efficiency by automatically learning from raw data and completing the 3D model more quickly. In addition, a large cohort of patients is required to validate the accuracy and safety of preoperative 3D reconstruction.

To solve the above problems, in a recent study of eBioMedicine, the researchers established a fully automated reconstruction system based on deep learning using a 3D convolutional neural network (CNN) and improved its performance by multi-task learning, attention mechanism, and deep supervision. After 500 training cases, the Dice similarity coefficient (validation metric to gauge the similarity of two samples) reached 89.2%. Finally, they compared the virtual broncho-vascular structure phantom to demonstrate its effectiveness with anatomical structures.

Comparing retrospective and prospective cohorts for accuracy was given. For safety verification, the retrospective cohort (without AI assistance) was compared with 128 prospective cohorts (with AI assistance). Finally, they evaluated the AI system's accuracy, quality score, and total reconstruction time against a manual reconstruction system. Based on the results of the retrospective and prospective cohorts, the proposed AI system proved accurate. The Safety verification of the AI system was measured using the operation time and intraoperative blood loss, and the result showed no significant influence on intraoperative blood loss. The efficiency verification of the AI system is measured through the accuracy comparison, quality scoring, and time consumption with Mimics© software.^6^

However, deep learning, in general, and convolutional neural networks (a popular type of deep learning), specifically in medical imaging analysis,^7^^,^^8^ are expected to continue to evolve. Thus, researchers working in this domain have a lot of challenges and ideas to address.

The lack of a large data set of medical images makes learning from small datasets of unlabelled images a useful research direction if an efficient model must be developed. Furthermore, developing an efficient transfer learning and data augmentation technique based on 3D CNN will help solve the need for more training datasets.

Through the discussion with some radiologists and GPs, I noticed the missing essential information that may lead to better model accuracy if we consider them while building the deep learning model.

Among the issues to be addressed are generalization (ability to deal with unseen data) and robustness (preserving model performance under naturally occurring image distortions or corruptions).

Explainability, explainability, and explainability need to be addressed, especially for medical image diagnostics. So radiologists can understand the system output analysis.

## Declaration of interests

S.K. has no conflict of interest to report.
